# Elimination of mutagenic contaminants from water using cellulose bearing ferrous-phthalocyanine

**DOI:** 10.1186/s41021-024-00317-5

**Published:** 2024-10-28

**Authors:** Kayoko Sano, Yuka Soga, Kaori Ohta, Yuki Kitamura, Sakae Arimoto-Kobayashi

**Affiliations:** 1 Graduate School of Medicine, Dentistry and Pharmaceutical Sciences, Okayama, 700-8530 Japan; 2 School of Pharmaceutical Sciences, Okayama University, Okayama, 700-8530 Japan

**Keywords:** Mutagen removal, Mutagen X, Contaminant elimination, Water chlorination, Water contaminants, Iron(ii) phthalocyanine, Ferrous phthalocyanine

## Abstract

**Background:**

We previously investigated methods for separating mutagenic contaminants from aqueous solutions using cellulose-bearing covalently bound trisulfo-Cu-phthalocyanine (blue cotton and blue rayon). Mutagenic contaminants with three or more fused aromatic rings in their structures were adsorbed onto blue cotton and rayon. Since Cu-phthalocyanine is considered an unsuitable absorption ligand for byproducts of water chlorination, such as 3-chloro-4-(dichloromethyl)-5-hydroxy-2(5H)-furanone (Mutagen X or MX), we investigated the development of a new material for the elimination of MX from aqueous solvents.

**Results:**

We selected green cellulose powder bearing ferrous phthalocyanine (FePh), hereafter referred to as green cellulose or GP, as the candidate material. GP is composed of cationized cellulose (white cellulose, WP) and FePh tetracarboxylic acid. The mutagenicity of MX dissolved in buffer or dimethyl sulfoxide (DMSO) solution significantly decreased after treatment with GP. The effects of GP on the elimination of MX from the solvent were very close to being expired after 70 cycles of repeated adsorption of the same GP, and the capacity of GP for MX removal was estimated to be exhausted after 120 cycles of repeated adsorption based on the extrapolation of the obtained result; thus, the interacting ligands on GP may be saturated after complete MX adsorption. The mutagenicity of MX dissolved in aqueous buffer significantly decreased after treatment at pH7.4 but not at pH 4.0. Since MX is dissociated to be the anionic form at pH 6 or higher, the negative charge of MX in the buffer at pH 7.4 may interact with the positive charge of ferrous ions in GP to create a linkage between MX and GP. After GP adsorbed MX, mutagenicity was extracted with water or acetonitrile and recovered in the eluent. Thus, the reversible interaction between MX and FePh may have caused adsorption of MX onto GP.

**Conclusion:**

GP could be used as a new eliminator and recovery agent for MX in chlorinated drinking water. Developing new materials for the removal and recovery of agents for the detection of mutagenic contaminant-related chlorination in water is beneficial for environmental health.

**Supplementary Information:**

The online version contains supplementary material available at 10.1186/s41021-024-00317-5.

## Introduction

Chemical pollution in environmental water is a severe health hazard, as groups of contaminants include products of chlorination [1], microplastics [2], polyaromatic hydrocarbons and nitroaromatic compounds [3, 4], *N*-nitrosamine [5], and heavy metals [6].

**Fig. 1:**
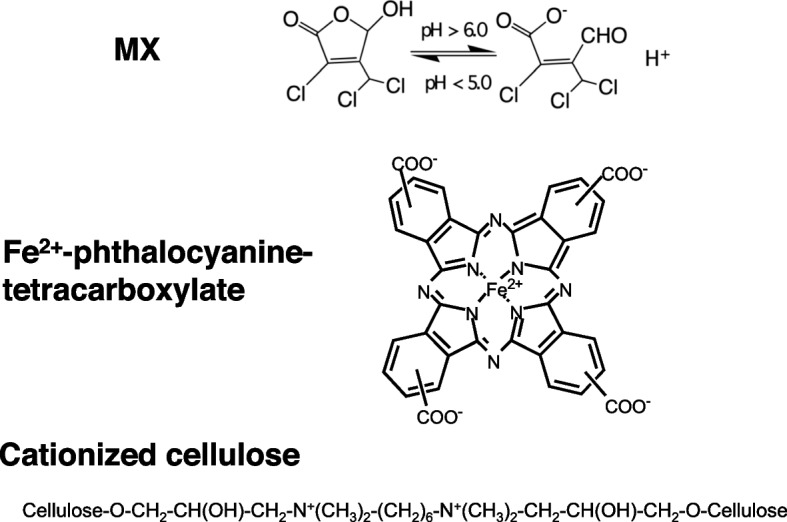
Structures of MX, Fe-phthalocyanine tetracarboxylate, and cationized cellulose

Previously, we investigated methods for the separation of chemical pollutants from their aqueous solutions using cellulose-bearing covalently bound trisulfo-Cu-phthalocyanine (blue cotton and blue rayon) [7]. Using blue cotton and rayon, chemicals with three or more fused aromatic rings in their structures (such as mutagenic chemicals found in the urine of smokers, cooked beef, and river water) are efficiently adsorbed [8, 9]. However, these methods are considered ineffective for the detection and removal of contaminants with two or fewer fused aromatic rings in their structure, such as 3-chloro-4-(dichloromethyl)-5-hydroxy-2(5H)-furanone (Mutagen X or MX), a byproducts of the disinfection of water by chlorination. MX is one of the strongest bacterial mutagens ever tested [10], with no rings in the structure at pH > 6.0 and one ring at pH < 5.0 in its structure (Fig. 1) [11].

We aimed to identify effective ligands for absorption and removal of MX from aqueous environments. Ferrous-phthalocyanine (FePh) tetracarboxylate, whose chemical structure is shown in Fig. 1, is a metallophthalocyanines. FePh nanopores exhibit promising potential for water desalination [12], and FePh derived products have been reported as molecular models for electrocatalytic oxygen reduction reactions [13]. FePh is a candidate of adsorption and removal material for MX.

In the present study, we developed a new material for the elimination and detection of mutagenic water-contaminants using FePh-fixed cellulose, and investigated its mechanisms.

## Materials and methods

### Materials

MX (CAS 77439–76-0, standard for water analysis), methyl methanesulfonate (CAS 66–27-3, MMS), 1-nitropyrene (CAS 5522–43-0), benzo[a]pyrene (CAS 50–32-8), and acetonitrile were purchased from FUJIFILM Wako Chemicals (Osaka, Japan). Dimethyl sulfoxide (DMSO) was purchased from Dojindo Laboratories (Kumamoto, Japan). For metabolic activation, the supernatant fraction of rat liver homogenate prepared using phenobarbital and 5,6-benzoflavone was obtained from FUJIFILM Wako Chemicals.

Cationized cellulose powder (white cellulose or WP) and cellulose powder bearing FePh-tetracarboxylic acid (green cellulose or GP) were solid powder obtained from Daiwabo Co., Ltd. (Osaka, Japan). WP was prepared as follows: a methylol moiety of polysaccharide unit in cellulose was linked covalently to a component with two molecules of quaternary ammonium cations, and then the component was covalently linked to another methylol moiety of the another polysaccharide unit, as shown in Fig. 1. GP was prepared using WP and FePh tetracarboxylates. The negative charge of the carboxyl moiety of FePh tetracarboxylate form an ionic linkage and are strongly connected to the cationic moiety of WP. FePh tetracarboxylate linked WP formed GP. The FePh content (mw. 740.4) in GP was 2% of cellulose (w/w %), thus approximately 20 μg (27 nmole) of FePh was contained in the 1 mg of GP. Preparation of WP and GP was outsourced to Daiwabo Co., Ltd. (Osaka, Japan).

*Salmonella enterica subspecies I, serovar Typhimurium* (*Salmonella typhimurium)* strain TA100 [*hisG46 ΔuvrB gal bio chl1005 rfa1001/pKM101*] and TA98 [*hisD3052 ΔuvrB gal bio chl1005 rfa1001/pKM101*] was provided by Dr. B. N. Ames from the University of California, Berkeley [14]. All the other reagents were purchased from commercial sources.

### Mutagen elimination from the solvents using GP

The powder dose-dependent change in regard to MX removal was investigated as follows; 0.4 mL of MX solution (MX 1 μM) dissolved in Na-phosphate buffer (10 mM, pH 7.4) was added to a test tube. WP or GP (solid powder 0–10 mg per 0.1 mL of MX solution, [0–40 mg / tube]) was added to the mixture. Tubes were shaken for 30 min, and then centrifuged at 10,000 rpm for 5 min at 4°C. Samples (0.1 mL) were collected from the supernatant, and assayed using the Ames test with *S. typhimurium* TA100 without metabolic activation [14]. The experiment was performed in triplicate.

Elimination depending on the MX concentration was performed as follows: A set of three tubes of MX solution (0–5 μM, 0.8 mL each) dissolved in Na-phosphate buffer (10 mM, pH 7.4) was prepared. The first tube was a no-powder control (NP), in which the powder was not added. GP (solid powder 10 mg / 0.1 mL of MX solution, [80 mg/tube]) was added to the second tube, and WP (solid powder 10 mg / 0.1 mL of MX solution, [80 mg/tube]) was added to the third tube. Tubes were shaken for 30 min, and then centrifuged at 10,000 rpm for 5 min at 4°C. Samples (0.1 mL) of each were taken from the supernatants and assayed using the Ames test with *S. typhimurium* TA100 without metabolic activation.

In addition to phosphate buffer, DMSO and acetonitrile were also examined as solvents. Three tubes of MX (0–5 μM, 0.8 mL each) dissolved in DMSO or acetonitrile were prepared. NP, GP (10 mg/ 0.1 mL) or WP (10 mg/ 0.1 mL) was added to the tubes, then tubes were shaken for 30 min, and centrifuged at 10,000 rpm for 5 min at 4°C. Samples (0.1 mL) were collected from the supernatants. Samples dissolved in DMSO were assayed using the Ames test as described above. Samples of acetonitrile solution were evaporated under reduced pressure to remove the acetonitrile, and the residues were dissolved in the same volume of water (0.1 mL) and assayed using the Ames test, as described above. The experiment was performed in triplicate.

The elimination of MMS (0–200 mM), 1-nitropyrene (0–0.02 mM), and benzo[a]pyrene (0–0.2 mM) from DMSO solution was also examined as described in the MX experiments. The mutagenic activity of MMS was assayed using the Ames test using *S. typhimurium* TA100 without metabolic activation, and that of 1-nitropyrene was assayed using *S. typhimurium* TA98 without metabolic activation. Benzo[a]pyrene was assayed using S*. typhimurium* TA100 with metabolic activation [14]. The experiment was performed in triplicate.

For the experiments examining the effects of pH, 1 mM sodium acetate buffer (pH 4.0) or 1 mM sodium phosphate buffer (pH 7.4) was used as the MX solvent. 0.4 mL of MX solutions (MX 0, 1 or 5 μM) dissolved in buffer (pH4.0 or pH 7.4) mentioned above were shaken for 30 min with GP (10 mg/ 0.1 mL), WP (10 mg/ 0.1 mL), or no-powder added (NP). The mixtures were centrifuged, and aliquots (0.1 mL, [MX 0, 0.1 or 0.5 nmole]) of the supernatant were collected and assayed using the Ames test using *S. typhimurium* TA100 without metabolic activation. The experiment was performed in triplicate.

For repeated experiments, three tubes with MX (10 μM, 0.4 mL each) dissolved in Na-phosphate buffer (20 mM, pH 7.4) were prepared. The first tube was used as a no-powder control. WP and GP (10 mg / 0.1 mL, each) were added to the second and third tubes, respectively. Tubes were shaken for 30 min, and then centrifuged at 10,000 rpm for 5 min at 4°C. Samples (0.1 mL) were collected from the supernatant and assayed using the Ames test. The number of revertants (/plate) obtained with the samples (0.1 mL, [1 nmole of MX]) from no-powder control was used as 100% control. The GP and WP precipitates obtained after centrifugation were used in the next cycle. The elimination experiment was repeated 70 times, and supernatant samples at first, second, third, 5th, 10th, 15th, 20th, 25th, 30th, 35th, 40th, 45th, 50th, 55th, 60th, 65th, and 70th cycles were assayed using the Ames test. The experiment was performed in triplicate.

### Recovery of mutagenicity from treated GP or WP

The recovery of mutagenicity from treated GP or WP was performed as follows. Tubes with MX solution (10 μM) dissolved in Na-phosphate buffer (20 mM, pH 7.4, 0.4 mL) were prepared. WP or GP (160 mg/tube) was then added. Tubes were shaken for 30 min, and then centrifuged at 10,000 rpm for 5 min at 4°C. The supernatant was removed. In the second adsorption cycle, a solution of MX (10 μM) in sodium phosphate buffer (20 mM, pH 7.4, 0.4 mL) was added to the tubes containing the precipitate (WP or GP). Tubes were shaken for 30 min, and then centrifuged as described above. The adsorption cycle was repeated for five times and precipitates (approximately 160 mg/tube) were obtained. To the tubes containing the precipitates, 1 mL of distilled water, acetonitrile, DMSO, methanol or methanol-conc. NH_4_OH (50: 1) was added as the eluent. Tubes were shaken for 30 min, and then centrifuged at 10,000 rpm for 5 min at 4°C. Samples (0.1 mL each) were collected from the supernatants, and the samples from the aqueous solution or DMSO were assayed using the Ames test, as described above. Samples (0.1 mL) obtained from the acetonitrile, methanol or methanol-conc. NH_4_OH (50: 1) evaporated under reduced pressure to remove the eluent, and the residues were dissolved in water (0.1 mL) and assayed using the Ames test as described above. Distilled water (0.1 mL) was used as a negative control (NC) in the Ames assay.

The variable conditions of different experiments are shown in supplemental table.

### Statistical analyses

Data were expressed as mean ± SD for each data point, as indicated in each figure. Error bars represented the SD. Statistical analyses were performed using KaleidaGraph (Synergy Software, Reading, PA, USA) and Excel statistics (SSRI Co. Ltd., Tokyo, Japan). Statistical significance was set at *p* < 0.05.

## Results

**Fig. 2:**
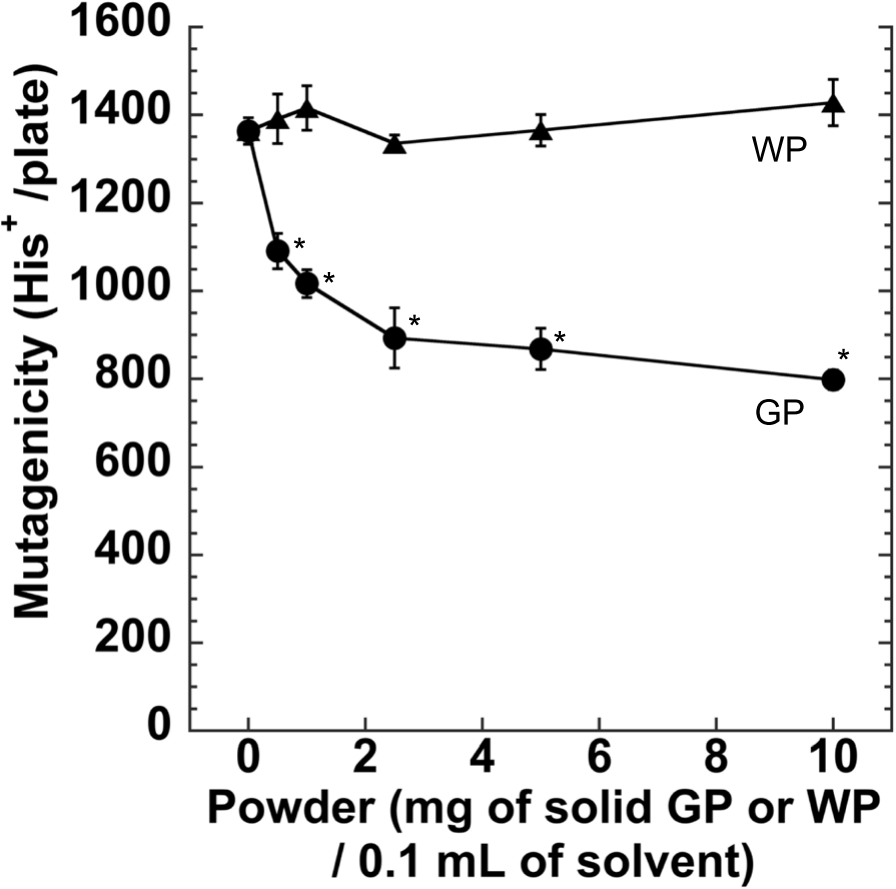
Mutagenicity of the supernatant (0.1 mL) obtained from MX solution (1 μM) treated with GP (circle) or WP (triangle). The amount of powder used for elimination (mg of solid GP or WP / 0.1 mL of solution) is indicated on the horizontal axis. The experiment was repeated twice, and the error bar represents the SD (*n* = 3). *: *p* < 0.05, significantly different from no-powder control

The mutagenicity of the MX solution was significantly decreased after the treatment with GP (0.5–10 mg) compared to that with no treatment with GP (no-powder control, 0 mg GP) in a GP-dose dependent manner (Fig. 2). Statistical analyses were performed using Dunnett's test. The mutagenicity of the supernatant did not decrease nor increase with the treatment with WP (0.5–10 mg) compared to that of the no-powder control (0 mg of powder) (Fig. 2). The mutagenicity remained in supernatant after GP treatment was 80.0 ± 3.0% with 0.5 mg GP in 0.1 mL, and 58.5 ± 1.6% with 10 mg GP in 0.1 mL, respectively. Thus, the adsorption % of MX solution (1 μM) was 20.0% with 0.5 mg GP in 0.1 mL, and 41.5% with 10 mg GP in 0.1 mL, respectively (Fig. 2). The number of spontaneous revertants/plate (NC) was 79 ± 7.48 using *S. typhimurium* TA100 without metabolic activation. We selected 10 mg / 0.1 mL as the dose of GP or WP for further experiments.

**Fig. 3:**
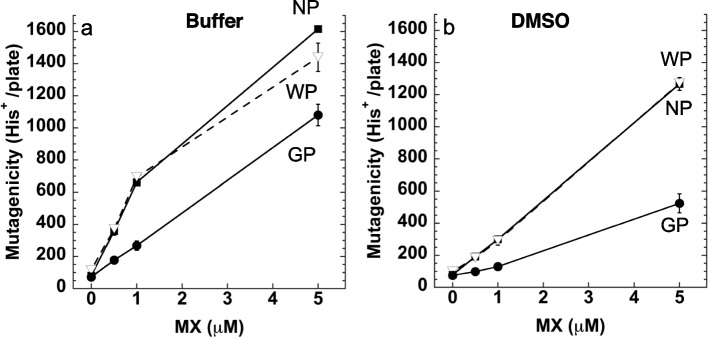
Mutagenicity of the sample (0.1 mL) of supernatant obtained from MX solution (0–5 μM) treated with 10 mg/0.1 mL of GP (circle) or WP (triangle, dashed line), or no-powder control (NP, square). MX was dissolved in buffer (**a**) or DMSO (**b**). The experiment was repeated twice, and the error bar represents the SD (*n* = 3)

The effects of MX concentration (0–5 μM) on the elimination efficiency were examined (Fig. 3). After the treatment with GP (10 mg / 0.1 mL), the mutagenicity of the supernatant of the MX solution (0.5–5 μM) dissolved in buffer was significantly decreased compared with those in the WP treatment or no-powder control (Fig. 3a). Treatment with WP in MX solution resulted in no decrease nor increase in the mutagenicity of the supernatant compared to the no-powder control. The mutagenicity of MX (0.5–5 μM) dissolved in DMSO also significantly decreased after GP treatment, but not after WP treatment, compared to the no-powder control (Fig. 3b). The mutagenicity of MX (0.5 μM) was 49.5 ± 4.0% removed from buffer and 51.1 ± 4.3% was removed from DMSO after treatment with GP (10 mg / 0.1 mL), respectively (Fig. 3). The mutagenicity of the supernatant of MX dissolved in acetonitrile did not decrease nor increase after GP nor WP treatment (data not shown). The mutagenicity of MMS, 1-nitropyrene, and benzo[a]pyrene dissolved in DMSO did not decrease or increase with GP nor WP treatment (data not shown). Statistical analyses were performed using Tukey's test. Further experiments were conducted using aqueous solutions of MX.

**Fig. 4:**
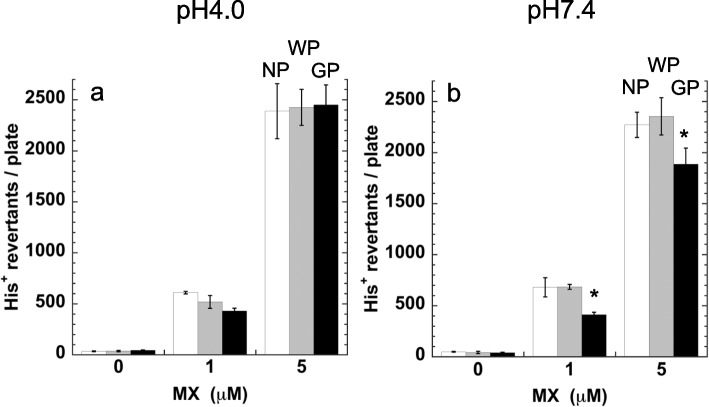
Mutagenicity of the sample (0.1 mL) of supernatant obtained from MX (0, 1, 5 μM) solution at pH 4.0 (**a**) or pH 7.4 (**b**) treated with no-powder control (NP, white) or WP (shaded) or GP (black). The experiment was repeated twice, and the error bar represents the SD (*n* = 3). *p < 0.05, significantly different from no-powder control and WP using Tukey test

We investigated the effects of the pH on the interaction between MX and the GP (Fig. 4). If the ionic interaction between MX and FePh participates in the adsorption of MX to GP, the adsorption efficiency of GP might differ from that in a neutral to acidic (pH < 5.0) solvent. When MX was dissolved in 1 mM sodium acetate buffer at pH 4.0, no significant difference in mutagenicity was observed among the samples treated with GP, WP or the no-powder control (Fig. 4a). As a positive control, the mutagenicity of MX dissolved in sodium phosphate buffer (pH7.4) was significantly decreased after the treatment with GP compared to that of the no-powder control or with WP (Fig. 4b).

**Fig. 5:**
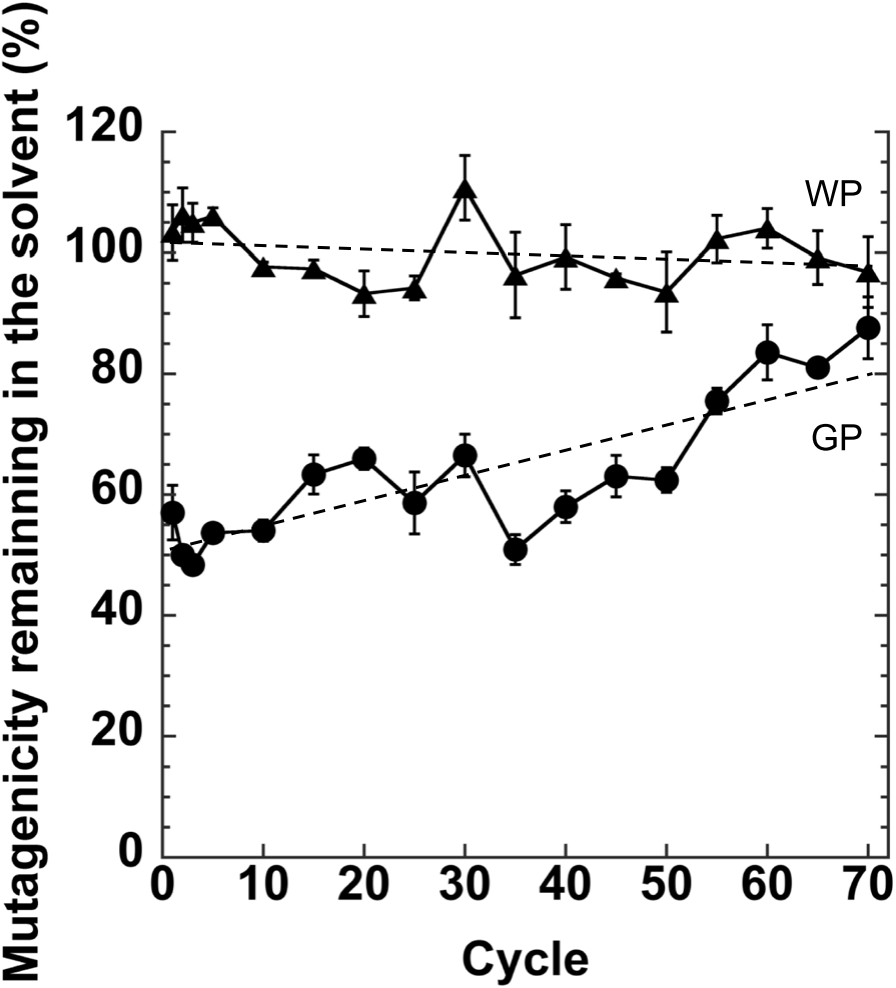
Mutagenicity (%) remaining in the supernatant after each treatment cycle with GP (circle) or WP (triangle). 100% was 626 ± 33.33 (His^+^ revertants / plate). The experiment was repeated twice, and the error bar represents the SD (*n* = 3). Regression lines are also shown (dashed line)

We investigated whether the elimination effect was caused by the adsorption of MX to GP, or by the chemical destruction and disappearance of MX on GP. If MX was adsorbed onto the GP ligand, saturation of the adsorption site with MX would be observed after most of the available ligands on the GP had been occupied by the MX molecules. The mutagenicity remaining in the solvent increased with repeated adsorption (Fig. 5). After adsorption was repeated 70 times, the mutagenicity remaining in the solvent treated with GP approached nearly 100% of that of the original solution (Fig. 5), which was 626 ± 33.3 (His^+^ revertant/plate). The regression line for the mutagenicity remaining in the GP-treated eluent was calculated as y = 50.225 + 0.42546 × R = 0.8316. Here, "y" is mutagenicity remaining in the solvent (%), and "x" is adsorption cycle. According to this approximate formula, the mutagenicity remaining in the eluent treated with GP would be 100% after 117 treatment cycle. The regression line for WP was calculated as y = 102.11–0.064723 × R = 0.29577.

**Fig. 6:**
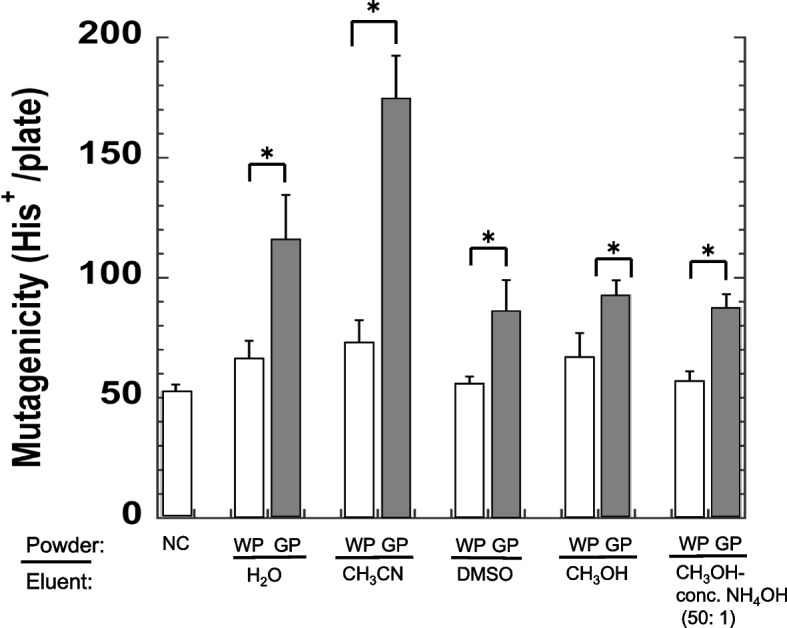
Recovery of the mutagenicity from GP or WP. NC was negative control of the Ames test. The experiment was repeated twice, and the error bar represents the SD (*n* = 3). *: *p* < 0.05, significantly different

If the elimination of MX from the solvent was due to adsorption but not the destruction by FePh in GP, a suitable eluent might recover the mutagenicity from treated GP. We investigated the adsorption and recovery of the mutagenicity from GP. After 5th cycle of adsorption of MX to GP or WP, the treated GP or WP was washed with the eluent, and the mutagenicity of the eluent was examined (Fig. 6). When the treated GP was washed with an eluent (water, acetonitrile, DMSO, methanol or methanol-conc. NH_4_OH (50: 1)), the mutagenicity of eluent (0.1 mL) was significantly increased when compared with NC (Fig. 6). A significant difference was observed between the mutagenicity of each eluents obtained from the GP and WP samples (Fig. 6). The mutagenicity of the GP sample eluent was highest with acetonitrile than with water, DMSO, methanol or methanol-conc. NH_4_OH (50: 1). The second best eluent was water. No increase in mutagenicity was observed in the eluent from the treated WP. Statistical analyses were performed using Tukey's test.

## Discussion

Water decontamination tools have been investigated for removing environmental contaminants in water, such as polyaniline-based adsorbents for the removal of hexavalent chromium (Cr (VI)) [15], natural and synthetic materials for fluoride contamination [16], bioflocculants for microplastics pollution [2], and coconut coir powder for dye‑contaminated wastewater [17]. Bagheban et al. developed graphene oxide-coated sand particles for the removal of MX from water [18], and the sand particles (80 g/L) decreased the concentration of MX from 20 μg/L (0.092 μM) to 2 μg/L under the optimum conditions.

We developed a new material for the elimination of mutagenic contaminants. Ideally, it would be desirable for a smaller amount of absorbent to remove MX at lower concentrations. Treatment with GP (0.5 mg/0.1 mL, i.e., 5 g/L) decreased the mutagenicity of MX (1 μM) dissolved in buffer by 20% (Fig. 2). One of the merits of GP is that a lower amount of adsorbent (GP 5 g/L) can remove MX from the solvent compared to that for Bagheban's graphene-coated sand (80 g/L) [18].

Since the content of FePH in GP was 13.5 nmole/0.5 mg GP, removed MX (20% of 1 μM of 0.1 mL, [0.02 nmole]) corresponds to the 0.15% of the total FePh. It is likely that a part of the FePh in GP was available for interaction with MX. GP eliminated the mutagenic activity of MX dissolved in water and DMSO (Fig. 3). Polar solvents may be appropriate for the interaction between MX and FePh in the GP. The percentages of MX (0.5–5 μM) removed was rather constant at approximately 50% of MX.

Since MX is dissociated to its anionic form at pH 6 or higher, it has a negative charge in Na-phosphate buffer at pH 7.4; however MX is not dissociated and has no charge at pH 4 (Fig. 1). As shown in Fig. 4, MX dissolved in the buffer at pH7.4 but not at pH 4.0, was eliminated by GP treatment. The negative charge of MX may interact with the positive charge of the ferrous ions in GP to form a linkage between MX and GP, and the ionic interaction between FePh and MX may cause the MX adsorption onto GP.

If GP disrupts MX to form nonmutagenic compounds, it is expected to be reusable. However, the effects of GP on the elimination of MX from the solvent were close to being expired after 70 cycles of repeated adsorption of the same GP, and the capacity of GP for MX removal was estimated to be exhausted after 120 cycles of repeated adsorption, based on the extrapolation of the obtained result (Fig. 5). This means that the available ligands on GP interacting with MX were saturated after adsorption. The MX adsorbed on the GP was recoverable (Fig. 6), and the interaction of MX with the GP was a reversible linkage rather than a rigid connection, such as a covalent bonds.

The elution efficiency was higher with acetonitrile than with water, DMSO, methanol or methanol-conc. NH_4_OH (50: 1) (Fig. 6). When the treated GP was washed with acetonitrile, the mutagenicity of eluent (0.1 mL) was significantly increased by 122.3 (His^+^ revertant /plate), compared with that of NC, which corresponded to 1223 (His^+^ revertant /plate) per total eluent (1 mL). Based on the data in Fig. 3a (no-powder control), 1223 (His^+^ revertant /plate) corresponded to the mutagenicity of approximately 3.5 μM MX. Then, about 3.5 nmole of MX was present in 1 mL eluent. After five adsorption cycles, maximum of 20 nmole of MX (0.4 mL of 10 μM, five times) was adsorbed onto the GP (160 mg). However, based on the experiment described in Fig. 4, the adsorption efficiency from first to 5th cycle was 43–52% (average 47 ± 3.4%). Therefore, approximately 9.5 nmole of MX was adsorbed. Then, elution efficiency was calculated to be about 37% (Fig. 6). Low recoveries of mutagenicity might be caused by the degradation of MX. However, the response to MX mutagenicity seems to vary from one experiment to another, and therefore the elution efficiency may also vary with the conditions. Further researches should be conducted in this regard.

An eluent with acetonitrile can be easily concentrated by evaporation, and GP may provide a separation and concentration methods for low amounts of MX in environmental waters. The formation of byproducts related to water chlorination, such as trihalomethanes and MX, in drinking water remains a severe problems for human health [19]. Thus, GP may be a candidate of eliminator and detection tools for MX in chlorinated drinking water. The development of this novel method for chlorination-related mutagenic contaminants from water presents potential for improving environmental water quality and public health.

Unfortunately, mutagenicity of MMS, 1-nitropyrene, and benzo[a]pyrene dissolved in DMSO did not decrease or increase after treatment with GP or WP. The elimination of these environmental contaminants is important for public health. Further development of tools and methods for contaminant removal is required.

## Supplementary Information

Supplementary Material 1.

## Data Availability

Data sharing is not applicable to this article.

## Abbreviations

White cellulose or WP: Cationized cellulose
blue cotton or blue rayon: Cellulose bearing covalently bound trisulfo-Cu-phthalocyanine
mutagen X or MX: 3-Chloro-4-(dichloromethyl)-5-hydroxy-2(5H)-furanone
DMSO: Dimethyl sulfoxide
FePh: Fe-phthalocyanine or ferrous-phthalocyanine
MMS: Methyl methanesulfonate
GP: Green cellulose
NC: Negative control
NP: No-powder control
Green cellulose or GP: Powder of cellulose bearing ferrous-phthalocyanine

## References

[1] Richardson SD, Plewa MJ, Wagner ED, Schoeny R, DeMarini DM. Occurrence, genotoxicity, and carcinogenicity of regulated and emerging disinfection by-products in drinking water: a review and roadmap for research. Mutat Res. 2007;636:178–242. 10.1016/j.mrrev.2007.09.001. 17980649 10.1016/j.mrrev.2007.09.001

[2] Muthulakshmi L, Mohan S, Tatarchuk T. Microplastics in water: types, detection, and removal strategies. Environ Sci Pollut Res Int. 2023;30:84933–48. 10.1007/s11356-023-28460-6. 37386221 10.1007/s11356-023-28460-6

[3] Vijayanand M, Ramakrishnan A, Subramanian R, Issac PK, Nasr M, Khoo KS, Rajagopal R, Greff B, Wan Azelee NI, Jeon BH, Chang SW, Ravindran B. Polyaromatic hydrocarbons (PAHs) in the water environment: a review on toxicity, microbial biodegradation, systematic biological advancements, and environmental fate. Environ Res. 2023;227:115716. 10.1016/j.envres.2023.115716. 36940816 10.1016/j.envres.2023.115716

[4] Bilal M, Bagheri AR, Bhatt P, Chen S. Environmental occurrence, toxicity concerns, and remediation of recalcitrant nitroaromatic compounds. J Environ Manage. 2021;291:112685. 10.1007/s11356-023-28460-6. 33930637 10.1016/j.jenvman.2021.112685

[5] Zhao B, Nakada N, Okumura K, Zhou J, Tanaka H. N-nitrosomorpholine behavior in sewage treatment plants and urban rivers. Water Res. 2019;163:114868. 10.1016/j.watres.2019.114868. 31344505 10.1016/j.watres.2019.114868

[6] Das S, Das S, Ghangrekar MM. Efficacious bioremediation of heavy metals and radionuclides from wastewater employing aquatic macro- and microphytes. J Basic Microbiol. 2022;62(3–4):260–78. 10.1002/jobm.202100372. 35014053 10.1002/jobm.202100372

[7] Hayatsu H, Oka T, Wakata A, Ohara Y, Hayatsu T, Kobayashi H, Arimoto S. Adsorption of mutagens to cotton bearing covalently bound trisulfo-copper-phthalocyanine. Mutat Res. 1983;119:233–8. 10.1016/0165-7992(83)90166-5. 6828061 10.1016/0165-7992(83)90166-5

[8] Hayatsu H, Hayatsu T, Arimoto S, Sakamoto H. A short-column technique for concentrating mutagens/carcinogens having polycyclic structures. Anal Biochem. 1996;235(2):185–90. 10.1006/abio.1996.0110. 8833326 10.1006/abio.1996.0110

[9] Arimoto-Kobayashi S, Lord GA, Hayatsu H. Mutagenicity in the surface waters from rivers in the UK and Japan from 1997 to 2005. Genes and Environment. 2007;29:67–73. 10.3123/jemsge.29.67.

[10] Meier JR, Knohl RB, Coleman WE, Ringhand HP, Munch JW, Kaylor WH, Streicher RP, Kopfler FC. Studies on the potent bacterial mutagen, 3-chloro-4-(dichloromethyl)-5-hydroxy-2(5H)-furanone: aqueous stability, XAD recovery and analytical determination in drinking water and in chlorinated humic acid solutions. Mutat Res. 1987;189:363–73. 10.1016/0165-1218(87)90044-9. 2960893 10.1016/0165-1218(87)90044-9

[11] Rincon E, Zuloaga F, Chamorro E. Global and local chemical reactivities of mutagen X and simple derivatives. J Mol Model. 2013;19:2573–82. 10.1007/s00894-013-1799-7. 23463265 10.1007/s00894-013-1799-7

[12] Deng Q, Pan J, Yin X, Wang X, Zhao L, Kang SG, Jimenez-Cruz CA, Zhou R, Li J. Toward high permeability, selectivity and controllability of water desalination with FEPC nanopores. Phys Chem Chem Phys. 2016;18:8140–7. 10.1039/c6cp00322b. 26923172 10.1039/c6cp00322b

[13] Kumar A, Ubaidullah M, Pandit B, Yasin G, Gupta RK, Zhang G. Fe-phthalocyanine derived highly conjugated 2D covalent organic framework as superior electrocatalyst for oxygen reduction reaction. Discov Nano. 2023;18:109. 10.1186/s11671-023-03890-w. 37665422 10.1186/s11671-023-03890-w PMC10477159

[14] Maron DM, Ames BN. Revised methods for the Salmonella mutagenicity test. Mutat Res. 1983;113:173–215. 10.1016/0165-1161(83)90010-9. 6341825 10.1016/0165-1161(83)90010-9

[15] Jiang Y, Liu Z, Zeng G, Liu Y, Shao B, Li Z, Liu Y, Zhang W, He Q. Polyaniline-based adsorbents for removal of hexavalent chromium from aqueous solution: a mini review. Environ Sci Pollut Res Int. 2018;25:6158–74. 10.1007/s11356-017-1188-3. 29307070 10.1007/s11356-017-1188-3

[16] El Messaoudi N, Franco DSP, Gubernat S, Georgin J, Şenol ZM, Ciğeroğlu Z, Allouss D, El Hajam M. Advances and future perspectives of water defluoridation by adsorption technology: a review. Environ Res. 2024;252:118857. 10.1016/j.envres.2024.118857. 38569334 10.1016/j.envres.2024.118857

[17] Bhattacharjee S, Kuila SB, Mazumder A. Surfactant-modified coconut coir powder (SMCCP) as a low-cost adsorbent for the treatment of dye-contaminated wastewater: parameters and adsorption mechanism. Environ Sci Pollut Res Int. 2024. 10.1007/s11356-024-34022-1[Online ahead of print]. 38904878 10.1007/s11356-024-34022-1

[18] Bagheban M, Mohammadi A, Baghdadi M, Janmohammadi M, Salimi M. Removal of mutagen X “MX” from drinking water using reduced graphene oxide coated sand particles. J Environ Health Sci Eng. 2019;17:827–37. 10.1007/s40201-019-00399-2. 32030156 10.1007/s40201-019-00399-2 PMC6985337

[19] Kothe A, Wachasunder N, Rodge A, Labhasetwar P, Maldhure A. Trihalomethanes in developed and developing countries. Environ Monit Assess. 2023;196(1):17. 10.1007/s10661-023-12106-8. 38057440 10.1007/s10661-023-12106-8

